# Meta-analysis of the effects of inert gases on cerebral ischemia–reperfusion injury

**DOI:** 10.1038/s41598-023-43859-4

**Published:** 2023-10-06

**Authors:** Di Wu, Daoyu Zhang, Hang Yin, Bo Zhang, Jihong Xing

**Affiliations:** 1 Department of Emergency Medicine, The First Hospital of Jilin University, No.71 Xinmin Street, Changchun, 130021 Jilin China; 2 The First Hospital of Jilin University, Changchun, 130021 Jilin China; 3 Baicheng Medical College, Baicheng, 137000 Jilin China; 4 The Second Foreign Department, Corps Hospital of the Chinese People’s Armed Police Force of Jilin Province, Changchun, 130052 Jilin China

**Keywords:** Cardiovascular diseases, Medical research

## Abstract

Recently, noble gas has become a hot spot within the medical field like respiratory organ cerebral anemia, acute urinary organ injury and transplantation. However, the shield performance in cerebral ischemia–reperfusion injury (CIRI) has not reached an accord. This study aims to evaluate existing evidence through meta-analysis to determine the effects of inert gases on the level of blood glucose, partial pressure of oxygen, and lactate levels in CIRI. We searched relevant articles within the following both Chinese and English databases: PubMed, Web of science, Embase, CNKI, Cochrane Library and Scopus. The search was conducted from the time of database establishment to the end of May 2023, and two researchers independently entered the data into Revman 5.3 and Stata 15.1. There were total 14 articles were enclosed within the search. The results showed that the amount of partial pressure of blood oxygen in the noble gas cluster was beyond that in the medicine gas cluster (P < 0.05), and the inert gas group had lower lactate acid and blood glucose levels than the medical gas group. The partial pressure of oxygen (SMD = 1.51, 95% CI 0.10 ~ 0.91 P = 0.04), the blood glucose level (SMD = − 0.59, 95% CI − 0.92 ~ − 0.27 P = 0.0004) and the lactic acid level (SMD = − 0.42, 95% CI − 0.80 ~ − 0.03 P = 0.03) (P < 0.05). These results are evaluated as medium-quality evidence. Inert gas can effectively regulate blood glucose level, partial pressure of oxygen and lactate level, and this regulatory function mainly plays a protective role in the small animal ischemia–reperfusion injury model. This finding provides an assessment and evidence of the effectiveness of inert gases for clinical practice, and provides the possibility for the application of noble gases in the treatment of CIRI. However, more operations are still needed before designing clinical trials, such as the analysis of the inhalation time, inhalation dose and efficacy of different inert gases, and the effective comparison of the effects in large-scale animal experiments.

## Introduction

Recently, the number of sudden death cases caused by cardiovascular diseases is increasing year by year, which is a growing concern^1,2^. Previous studies showed asystole was presently the leading reason behind death in patients with extra time^3^. At present, the current key to resuscitating cardiac arrest (CA) patients is the timely implementation of cardiac resuscitation (CPR) once CA occurs to make reperfusion in various organs of the patient during ischemia^4^. However, several studies have shown that after performing CPR on patients who experience cardiac arrest, most patients exhibit varying degrees of neurological deficits^5^, such as convulsions, coma, persistent vegetative state, and even death^6^, which is post-cardiac arrest syndrome (PCAS), this condition contains a poor prognosis and high mortality^7^. Tissue hypoperfusion and ischemia often occur in critically ill patients, and they are important factors contributing to multiple organ failure and perioperative mortality in patients. Therefore, ischemia–reperfusion injury (IRI) is a significant problem, with high incidence and mortality rates associated with various diseases it causes. This process involves a chain reaction of events that includes the activation of apoptotic pathways, inflammatory response, release of oxygen radicals, brain cell swelling, Ca^2+^ overload, and accumulation of excitatory amino acids (EAA) etc*.*^8,9^. Although there is currently no effective treatment for IRI, a growing number of studies are exploring the use of inhaled drugs to reduce IRI.

There have been studies on the use of two inert gases, helium and xenon, for the treatment of IRI. Helium is occasionally used for ventilatory therapy in patients with chronic obstructive pulmonary disease and for improving cerebral ischemia–reperfusion injury^10,11^. Xenon exhibits anesthetic properties under normobaric conditions and is known to be the fastest-acting and fastest-recovering among all known anesthetics. It also has highly desirable cardiovascular characteristics, making it very safe. Xenon also plays a certain role in brain protection during the middle and late stages of cerebral ischemia–reperfusion^12,13^. However, currently, there is no comprehensive review that summarizes and evaluates these studies. Therefore, this study aims to systematically review the literature to assess the current evidence on the use of inert gases for treating IRI and to explore the prospects and challenges of this treatment approach.

## Result

### Literature search results

**Figure 1:**
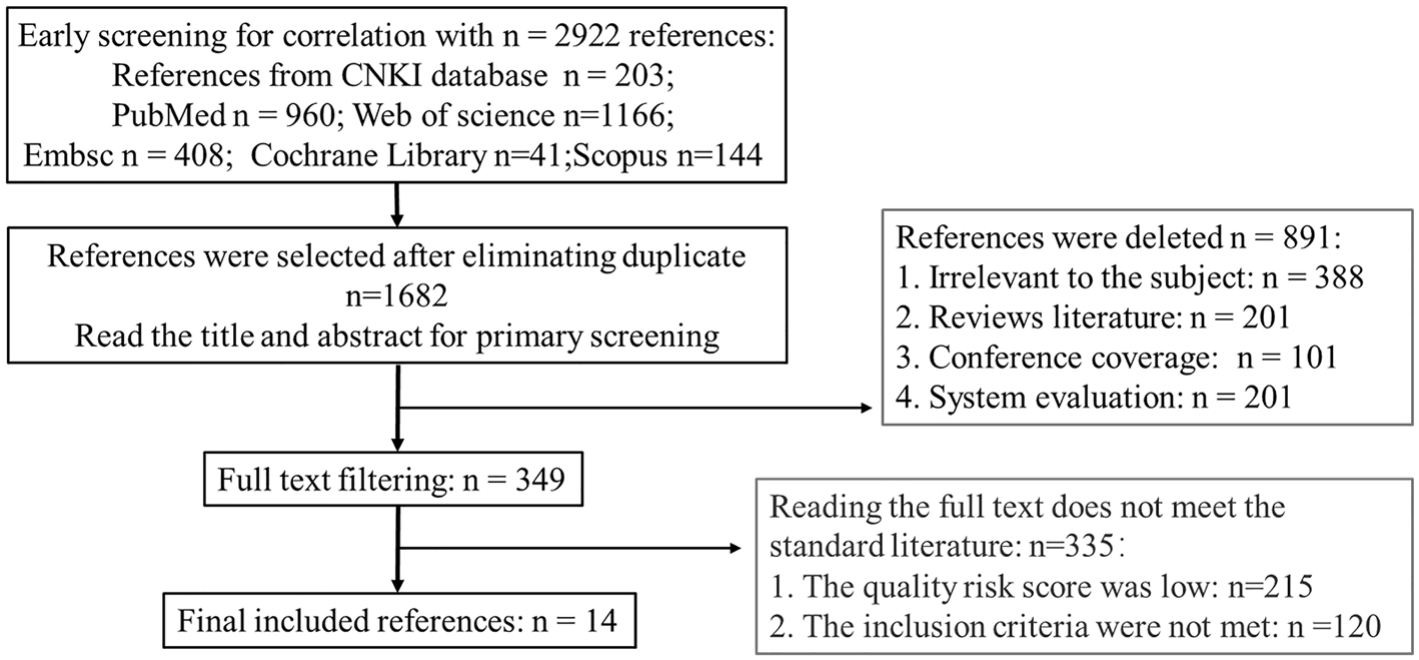
Flow diagram of study selection.

A total of 2292 articles were searched. 1240 duplicate studies were initially excluded, 891 studies were excluded (388 articles unrelated to the theme; 201 reviews; 101 conference reports; 25 systematic evaluations) after reading the title, 501 studies were excluded after reading the full text (21 papers with lower quality risk scores; 201 articles that do not meet the inclusion criteria). Therefore 14 articles were eventually included in this study. The literature selection process is shown in Fig. 1.

### General research features

This study included 14 relevant literatures published between 2003 and 2023. All interventions are based on inert gases. The measurement results include blood glucose levels, lactate acid levels, and partial pressure of oxygen. The basic characteristics of the included studies were listed in Table 1.

**Table 1:** Characteristics of studies included in the meta-anal.

Author (year)	Country	Design	Participants	Experimental group	Control group	Outcomes (Scale)
			(Exp/Con)			
Brücken (2014)	Germany	RCT	9/9	Noble gas (Argon)	Medical air	Glucose; Lactat; PaO_2_
Brücken (2017)	Germany	RCT	7/7	Noble gas (Argon)	Medical air	Lactat; PaO_2_
Liu (2019)	Germany	CCT	8/7	Noble gas (Argon)	Medical air	PaO_2_
Aehling (2017)	Germany	RCT	6/6	Noble gas (Helium)	Medical air	PaO_2_
Elavazhagan(2010)	United Kingdom	RCT	18/18	Noble gas (Xenon)	Medical air	Lactat
Helene (2007)	France	CCT	5/8	Noble gas (Xenon)	Medical air	PaO_2_
Helene (2012)	France	RCT	14/14	Noble gas (Argon)	Medical air	PaO_2_
Faulkner (2011)	United Kingdom	CCT	7/9	Noble gas (Xenon)	Medical air	Glucose; Lactat; PaO_2_
Benait (2016)	France	RCT	5/5	Noble gas (Helium)	Medical air	PaO_2_
Homi (2003)	USA	RCT	21/21	Noble gas (Xenon)	Medical air	Glucose; PaO_2_
Ryang (2011)	Germany	RCT	11/11	Noble gas (Argon)	Medical air	PaO_2_
Schmidt (2005)	Germany	RCT	12/12	Noble gas (Xenon)	Medical air	Glucose;Lactat;PaO_2_
Sheng (2012)	USA	RCT	13/12	Noble gas (Xenon)	Medical air	PaO_2_
Grüne (2017)	Netherlands	CCT	29/29	Noble gas (Argon)	Medical air	Glucose

### Evaluation of research quality

Research quality was scored from 3 to 6, and all included studies were published in peer-reviewed journals, 8 studies mentioned temperature control^14–21^: including room temperature or indoor water temperature; 9 studies used randomization^14–17,19–23^; 9 studies mentioned compliance with animal welfare regulations^14,15,18–24^; 11 studies stated that the use of anesthetics has no apparent neuroprotective properties^14–17,20–26^. 4 studies used animal models of diabetes, hypertension or geriatrics^18,19,26,27^. The quality assessment of the studies was shown in Table 2.

**Table 2:** Methodological quality assessment of the included Studies.

Study	1	2	3	4	5	6	7	8	9	10	Score
Brücken (2014)	√	√	√	UN	UN	√	UN	UN	√	√	6
Brücken (2017)	√	√	√	UN	UN	√	UN	UN	√	√	6
Liu (2019)	√	UN	UN	√	UN	√	UN	UN	UN	√	4
Aehling (2017)	√	√	√	UN	UN	√	UN	UN	UN	UN	4
Elavazhagan (2010)	√	√	√	UN	UN	√	UN	UN	UN	UN	4
Helene (2007)	√	√	UN	UN	UN	UN	√	UN	√	UN	4
Helene (2012)	√	√	√	UN	UN	UN	√	UN	√	UN	5
Faulkner (2011)	√	UN	UN	UN	UN	√	√	UN	UN	UN	3
Benait (2016)	√	√	√	UN	UN	√	UN	UN	√	UN	5
Homi (2003)	√	UN	√	UN	UN	√	UN	UN	√	UN	4
Ryang (2011)	√	√	√	UN	UN	√	UN	UN	√	UN	5
Schmidt (2005)	√	UN	√	UN	UN	√	UN	UN	√	UN	4
Sheng (2012)	√	UN	UN	UN	UN	√	UN	UN	√	UN	3
Grüne (2017)	UN	UN	UN	√	UN	√	UN	UN	√	UN	3

(1) Peer-reviewed journal. (2) Temperature control. (3) Animals were randomly allocated. (4) Blind established model. (5) Blinded outcome assessment. (6) Anesthetics used without marked intrinsic neuroprotective properties. (7) Animal model (diabetic, advanced age or hypertensive). (8) Calculation of sample size. (9) Statement of compliance with animal welfare regulations. (10) Possible conflicts of interest. UN is Unclear.

### Effectiveness of the intervention

#### The blood glucose levels

**Figure 2:**
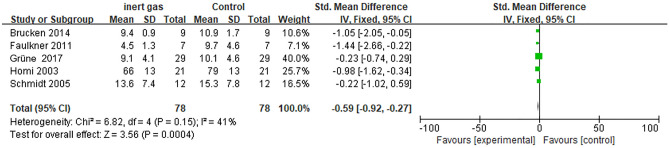
The forest map of blood glucose level: inert gas vs control.

Four existing studies reported that the levels of blood glucose showed statistically significant differences (P < 0.0001) between the intervention group (inert gas) and the control group (medical air) in animal models, the blood glucose level was significantly increased in the inert gas compared to medical air. Moreover, the level of heterogeneity among the 4 studies was relatively low (I^2^ = 17%, P = 0.31). Therefore, we used fixed effects model to analyze the data, as shown in Fig. 2 (SMD = − 0.59, 95% CI − 0.92 ~ − 0.27, P < 0.0001). The results of the fixed effect model obtained were SMD = 0.73, 95% CI 0.17 ~ 1.29 P = 0.01 after comprehensively removing a larger sample. This result showed there were no significant effects on the results when the model changed, indicating that the results of this meta-analysis were robust.

#### Lactic acid levels

**Figure 3:**
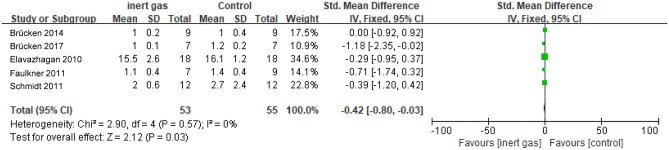
The forest map of the lactic acid level: inert gas vs control.

We observed differences in lactic acid levels between the intervention group (inert gas) and the control group (medical air). The analysis showed heterogeneity (I^2^ = 0%, P = 0.57) and used a fixed effect model (used a fixed-effects model) (Fig. 3). The effects confirmed that the level of lactic acid in the inert gas group was once decreased than that in the medical air group. The effects are proven in the determine (SMD = − 0.42, 95% CI − 0.80 ~ − 0.03, P = 0.03). After comprehensively putting off a giant sample, the consequences of the constant impact mannequin (SMD = − 0.49, 95% CI − 0.96 ~ − 0.01, p = 0.05) are obtained. The exchange of the mannequin has no tremendous effect on the results, indicating that the consequences of the meta-analysis were robust.

#### Partial pressure of blood oxygen

**Figure 4:**
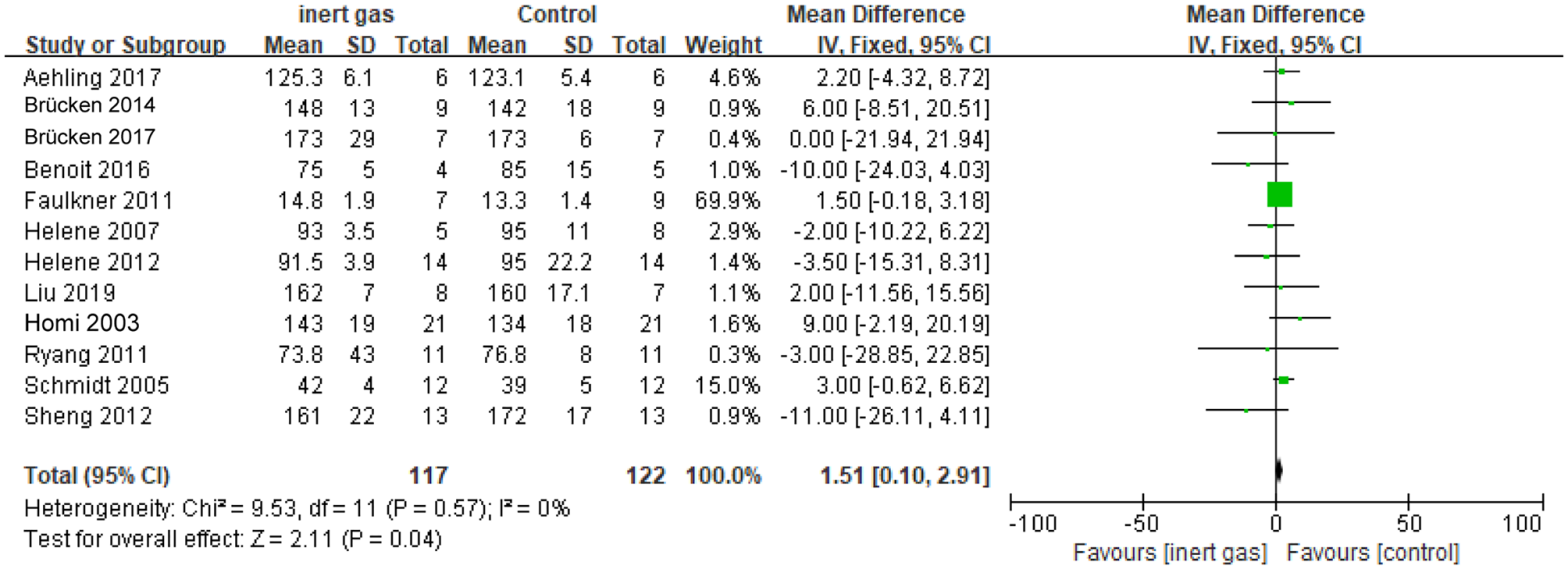
The forest map of blood oxygen partial pressure level: inert gas vs control.

There were differences in blood oxygen partial pressure between the inert gas group and the medical gas group. The results are shown in the Fig. 4 (MD = 1.51, 95% CI 0.10 ~ 2.91, P = 0.04). Heterogeneity was shown (I^2^ = 0%, P = 0.57) and a fixed effect model was used. The consequences confirmed that the partial stress of blood oxygen in inert gas group was once greater than that in the medical air group. After comprehensively doing away with a giant sample, the outcomes of the constant impact mannequin (MD = 1.50, 95% CI 0.09 ~ 2.91, p = 0.04) are obtained. The alternative of the mannequin has no full-size effect on the results, indicating that the effects of the meta-analysis were robust.

### Publication bias

Results checked by funnel plot and Egger's test showed no publication bias, the blood glucose (p = 0.585), partial pressure of oxygen (p = 0.336) and lactic acid (p = 0.230).

## Discussion

This study analyzed 14 published animal models of cerebral ischemia–reperfusion injury (CIRI), and compared the degree of cerebral ischemia–reperfusion injury (cerebral ischemia) and its blood glucose level, partial pressure of blood oxygen and lactate acid level in the animal models with inert gas inhalation and medical air inhalation. It is important to note that in animal models, the use of inert gas inhalation was found to be beneficial for controlling cerebral ischemia–reperfusion injury (brain ischemia) in terms of the blood glucose level, blood oxygen partial pressure and lactate acid level.

As is well known, the human brain relies on aerobic glycolysis to meet its metabolic needs, and it itself does not have any energy reserves^24^. Studies have shown that in the absence of energy in brain tissue, the transmembrane transport of glucose may be the rate-limiting process of glucose utilization. The body can attempt to ensure the most basic energy demand by increasing glucose uptake rate, which is a protective response of cells^28^. The results of this study indicate that in the early stages of cerebral ischemia–reperfusion injury (cerebral ischemia) in animal models, the use of inert gas inhalation results in stable and relatively non-high levels of glucose control, which is more conducive to brain protection. This is consistent with the view of some researchers that elevated blood glucose concentrations on admission to hospital in patients with cerebral ischemia–reperfusion injury are often associated with adverse outcomes in clinical practice, regardless of diabetes. At the same time, strict glycemic control in clinical practice has failed to produce any beneficial results^29,30^. After cerebral ischemia–reperfusion, hyperglycaemia is an independent risk factor for worsening prognosis, and the mechanism by exacerbates ischaemia/reperfusion injury may be directly related to elevated blood glucose, particularly neuroinflammation^31^. At the same time, preclinical studies in various animal I/R models and clinical studies to control elevated blood glucose have failed to produce neuroprotective effects, but have occasionally led to side effects^32,33^. Other studies have shown that an increase in glucose in the residual blood flow to the ischemic brain is beneficial for cell survival, as the reduced transport of glucose and oxygen during cerebral ischemia–reperfusion leads to significant depletion of adenosine 5 '- triphosphate (ATP) in the brain, which affects many downstream biological processes and leads to neuronal cell death^34,35^. In summary, blood glucose regulation is stable and maintained at a non-high level, which is more favorable for the recovery of cerebral ischemia–reperfusion injury.

In addition, the accumulation of lactic acid after cerebral ischemia leads to a decrease in the pH value of tissue, which can cause brain tissue damage, lipid peroxidation and free radical generation, eventually leading to irreversible cell damage. Nilsson et al. found that increased lactate levels are closely related to symptoms of cerebral ischemia, and high concentrations of lactate have a certain correlation with severe ischemia^36^. The lactate acid level in the brain during ischemia will not show a higher level when the glycogen reserve in the brain is very low, but during the period of enhanced anaerobic glycolysis, the lactate concentration will further increase. The production of brain lactate is mainly concentrated in the low perfusion period, accompanied by the increase of lactate, and irreversible damage to the structure of the cortical nerve cell membrane occurs. A study has reported that elevated glutamate induced by cerebral ischemia can lead to an increase in lactate production in astrocytes^37^. Persson et al. reported there was a threshold-type relationship between lactate concentration and glucose level^38,39^. Consistent with previous research findings, our study found the level of lactic acid was lower in the inert gas group compared to the control group at the early stage of CIRI, indicating the use of inert gas reduced the production of lactic acid, thus attenuating the occurrence of brain injury during ischemia–reperfusion.

Moreover, during periods of cerebral hypoperfusion, arterial oxygen pressure (PaO_2_) levels may be altered, which may affect the oxygen supply to the brain, thereby exacerbating the severity of brain injury. Elmer et al. confirmed that a threshold of 40 kpa for hyperoxia was an adverse outcome, but also suggested that moderate hyperoxia (PaO_2_ 13.5–39.9 kPa) levels may have beneficial effects on the recovery of brain injury^40^. When cerebral ischemia causes low partial pressure of oxygen (PaO_2_), a large increase in PaCO_2_ levels may be associated with worsening cerebral edema, respiratory acidosis, and impaired right ventricular function, all of which may lead to a poor prognosis^41–43^. While, studies have shown that high levels of PaO_2_ in the early stage of reperfusion after cardiac arrest can exacerbate ischemia–reperfusion injury^44^. They showed poor neurological prognosis and increased nerve damage after hyperoxemia^44^, this finding has also been confirmed by clinical human studies^45–47^. But in retrospective and observational studies in human, severe hyperoxemia (PaO_2_ > 40 kpa) was associated with a poor prognosis after cardiac arrest^48^. In contrast, moderate-to-moderate hyperoxia in intensive care after resuscitation was associated with better long-term neurological recovery and improved organ function. Retrospective analysis of a large ICU showed that the lowest mortality rate was associated with a PaO_2_ of approximately 20 kPa^49^. Therefore, an appropriate increase in partial pressure of oxygen (PaO_2_) can help the recovery of cerebral ischemia–reperfusion injury. Currently, there is limited high-quality data, and intervention studies are needed to determine the optimal oxygen concentration after cerebral ischemia and the potential association between oxygen therapy and prognosis.

Preclinical efficacy experiments are commonly cited to demonstrate the rationality of starting clinical trials. Our findings contribute to the literature on preclinical design and strengthen the exploratory study of the protective mechanism of inert gases in cerebral ischemia. Through this study, we determined that the induction of inert gases increased the animal's partial pressure of oxygen, proper blood glucose levels and decreased lactic acid level, which greatly reduced the degree of CIRI. Furthermore, this study can reduce unnecessary repeated experiments, facilitate deeper research in animal experiments, and may improve the success rate of future clinical trials.

However, the average quality score of the relevant studies during this paper is 4.38. Several studies don't describe their methods intimately, such as allocation concealment, blinded outcome assessment, hidden allocation etc. Additionally, there were several reasons that led to some biases: Firstly, different animal species, different concentrations, and different inhalation times were employed in various studies. Secondly, the animal models employed in most studies were healthy, while patients with cerebral ischemia–reperfusion injury (cerebral ischemia) usually suffer from polygenic disease, cardiovascular disease, hyperlipoidaemia and different connected underlying diseases. Thirdly, our search strategy only included Chinese and English databases. Fourth, the number of included literatures is limited, and sub-group analysis was not conducted. Therefore, the interpretation of this result ought to use caution.

In addition, heterogeneity in meta-analyses was influenced by experimental conditions tested in various original studies and differences in experimental settings, and may vary considerably. For example, in the experimental design of individual studies, according to most of the current studies, the most effective time point for inert gas administration is still unknown. In particular, pretreatment may be ideal for predicting the onset of Ischemia–reperfusion in surgical management, including brain, heart, kidney, liver, and even transplantation, However, the onset of ischemia is most often sudden or unexpected. Therefore, the conclusions of our meta-analysis should be interpreted as a simple summary of the results of the individual literature, rather than as a reference to the size of the expected effect in a well-defined homogeneous environment. While most of the available evidence confirms the role of inert gases in animal models of cerebral ischemia, it remains unknown whether the doses of brain protection found in animal experiments can be applied universally to the human environment. Different species show different sensitivities to inert gases, which may also be organ-specific.

## Conclusion

In summary, the results of this study indicate that the application of inert gases has a significant protective effect on experimental animals in cerebral ischemia–reperfusion injury (cerebral ischemia). Moreover, inert gases can alleviate ischemic brain injury by regulating blood glucose at a stable or non-elevated levels, increasing partial pressure of oxygen, and reducing lactate (salt) levels. However, the types of animals used and the methods of measuring results are all based on animals, which may have some deviation with future clinical trials. Therefore, consideration should be given to future clinical trial design and research. And inert gas will play a greater role in clinical applications in the future.

## Methods

Since all the studies included in this study are published articles, there are no ethical issues to disclosure.

### Retrieval strategy

This study was conducted according to the Preferred Reporting Items for Systematic Reviews and Meta-Analyses (PRISMA) and the PRISMA extension for Scoping Reviews. The retrieval database embraces English databases like PubMed, EMBASE, Web of Science, Cochrane Library, Scopus, and Chinese databases like CNKI. The retrieval time was from the establishment to May 31st, 2023. The search strategy was decided based on Pubmed's grid words combined with keywords from the necessary English and Chinese articles. English search terms include: (“cerebral anemia” or “cerebral schemia-reperfusion injury ”), (“inert gas” or “noble gas” or “helium ” or “xenon” or “neon”), (“Protective factor” or “Influencing factors ” or “Facilitator”) and (“randomized controlled trial” or “randomized” or “randomly” or “trial” or “groups”). The Chinese keywords include: (“cerebral anemia-reperfusion injury” or “cerebral ischemia, nerve injury” or “cerebral ischemia”), (“inert gas” or “rare gas” or “xenon” or “helium” or “neon” or “argon”), (“protective effect” or “influencing factor” or “promoting factor”) and (“randomized control” or “randomized grouping” or “randomizaton”).

After a literature search, we analyzed the titles and abstracts of the articles and excluded those that were not relevant to this meta-analysis. Next, we carefully read the full text of the remaining articles until all included articles were identified. In addition, to ensure the comprehensiveness of the search, we further searched the references of relevant reviews, meta-analyses or systematic reviews. All retrieved records are imported into Endnote X9 software for classification. After reviewing the titles and abstracts and reading the full text of the remaining studies, we selected studies that met the criteria for this meta-analysis. This process was completed independently by two researchers. In the case of different opinions, the decision was made after consultation with the third researcher.

This study was conducted according to the Preferred Reporting Items for Systematic Reviews and Meta-Analyses guidelines. It has been registered with the National Institute for Health Research in the International Prospective Register of Systematic Reviews (PROSPERO) (CRD42023457851).

### Inclusion and exclusion criteria

Inclusion:(1) The study subjects include animal models and humans; (2) The intervention group was treated with an inert gas for inducing cerebral ischemia–reperfusion injury, while the other group was treated with medical air for inducing cerebral ischemia–reperfusion injury; (3) The research content includes the determination of blood glucose level, partial pressure of oxygen and lactate (salt) level.

Exclusion: (1) Observational studies, reviews, comments, letters and conferences, literature with incomplete research data; (2) Repeat; (3) Full text not available; (4) The data is incomplete or not suitable for meta-analysis; (5) Other types of interventions are used besides inert gases.

### Data analysis

We extracted authors, years, countries, study design, sample size (experimental group/control group), participants, intervention, results etc*.* from the article. Then enter the data into Revman5.3 and STATA15.1. If I^2^ < 50%, P > 0.10, indicating low heterogeneity among included studies, a fixed-effects model was used. If I^2^ > 50%, P < 0.10, indicating that the included studies were highly heterogeneous, the results were summarized using a random-effects model. For continuous data, standardized mean difference (SMD) and mean weighted mean difference (MD) were used to calculate the corresponding 95% confidence interval (95% CI). When p < 0.05, the difference was statistically significant. Publication bias was assessed by visual funnel plots and quantitative computational Egger's test (STATA software).

### Risk of bias assessment

This study was assessed according to the ten-item checklist of the CAMARADES checklist: (1) Peer-reviewed journals; (2) Body temperature control; (3) Animals are randomly assigned; (4) Blind model building; (5) Blinded result assessment; (6) Use of anesthetics with no apparent neuroprotective properties Use of anesthetics with no apparent neuroprotective properties; (7) Animal model (diabetes、old age or high blood pressure); (8) Calculation of sample size; (9) Statement of compliance with animal welfare regulations; (10) Possible conflict of interest.

The quality of each study was assessed on a scale of 0 to 10. Data were extracted independently by two assessors and the quality of each study was assessed. In case of any discrepancies, it shall be resolved through discussion with a third person.

## Data Availability

The data that support the findings of this study are available in the methods.
